# Hidden Blue Hazard? LED Lighting and Retinal Damage in Rats

**DOI:** 10.1289/ehp.122-A81

**Published:** 2014-03-01

**Authors:** Tim Lougheed

**Affiliations:** Tim Lougheed has worked as a freelance writer in Ottawa, Canada, since 1991. A past president of the Canadian Science Writers’ Association, he covers a broad range of topics in science, technology, medicine, and education.

The Canadian government greeted 2014 with the first tier of its new energy-efficiency standards for light bulbs,^1^ which will effectively ban incandescent light bulbs by next year.^2^ But efficient lighting can have its own drawbacks. For instance, although devoid of the mercury used in compact fluorescent lamps (CFLs), some white light-emitting diodes (LEDs) emit a wavelength of light associated with adverse human health effects. In this issue of *EHP*, researchers study retinal changes in rats exposed to white LEDs like those sometimes used in household lighting.^3^

Among the most popular household LEDs are products that employ a chip emitting blue light, which is surrounded by a yellow phosphor coating. Although the resulting light looks white to the naked eye, it can feature a spike in the blue end of the spectrum, at wavelengths of 460–500 nm.

Light of this wavelength has been shown to have unique physiological effects, some positive, some negative.^4^^,^^5^^,^^6^ White LEDs, as a new source of exposure to blue light, initially prompted concerns about potential changes in melatonin production and disruption of human sleep cycles.^7^ More recent research considers the direct effect of this light on the eye, including the risk of ongoing damage to retinal cells.^8^

In the current study, the researchers wanted to accurately simulate exposure to indoor lighting, says corresponding author Chang-Ho Yang, a professor and ophthalmologist at National Taiwan University’s College of Medicine. He points out that earlier work shone light directly into the eyes of experimental animals, which may induce damage but hardly corresponds to the indirect way in which most people are exposed to artificial lighting.

“We created an exposure environment where rats could run freely in a cage with the light source set on the rack ceiling twenty centimeters above the cage roof,” Yang explains. “This mimics the ‘domestic lighting’ condition as much as possible, which should greatly reduce the injury—theoretically.”

**Figure:**
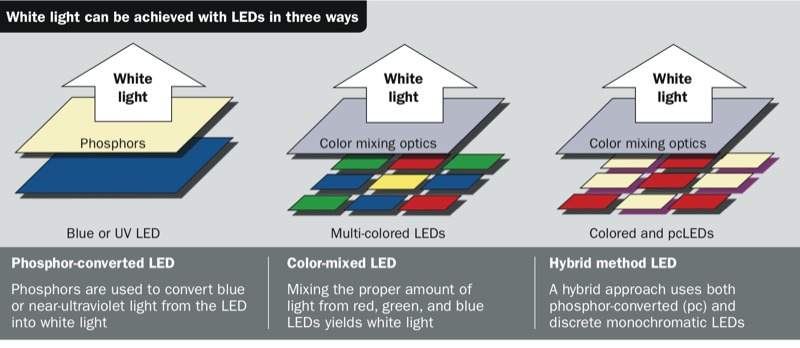
White light can be achieved with LEDs in three ways Source: Office of Energy Efficiency & Renewable Energy, U.S. Department of Energy; https://www1.eere.energy.gov/buildings/ssl/sslbasics_ledbasics.html

However, the retinas of rats exposed to either blue or cool white^9^ LED light showed evidence of retinal damage and cell death after 9 days of exposure. Although rats exposed to cool or warm white^10^ CFL lights also showed some evidence of damage relative to unexposed controls, in general differences were much less pronounced than those observed in the LED-exposed rats. The authors suggest the observed injuries may have been a consequence of oxidative stress from reactive oxygen species that were generated in retinal tissue.^3^

The rats used in these experiments were albino, and their unpigmented eyes were more sensitive to all effects of light. But even in typically pigmented eyes, Yang says, neuronal cells are incapable of repairing themselves or regenerating after damage. This makes it important to pin down mechanisms of injury and link them with clinical studies matching the conditions under which people will ultimately be using LED lighting. Future studies may suggest a spectrum threshold that could help the lighting industry optimize eye-friendly products, he notes.

Blue light is not without its virtues, says Seang Mei Saw, a professor of epidemiology and ophthalmology at the National University of Singapore, whose work deals with the onset of myopia in children. Time outdoors has shown a strong protective effect against myopia,^11^ and although the reasons are still undetermined, it’s possible blue light may play a role, Saw says—a hypothesis supported by evidence from animal studies.^12^ “Outdoor sunlight has more blue light that may protect for myopia,” she explains, “and indoor lighting, with relatively less blue light, may be detrimental for myopia.”
